# Behaviourally mediated predation avoidance in penguin prey: *in situ* evidence from animal-borne camera loggers

**DOI:** 10.1098/rsos.171449

**Published:** 2018-08-22

**Authors:** Jonathan M. Handley, Andréa Thiebault, Andrew Stanworth, David Schutt, Pierre Pistorius

**Affiliations:** 1 DST/NRF Centre of Excellence at the FitzPatrick Institute of African Ornithology, Department of Zoology, Nelson Mandela University, South Campus, Port Elizabeth 6031, South Africa; 2 Falklands Conservation, PO Box 26, Stanley FIQQ 1ZZ, Falkland Islands; 3 Department of Integrative Biology, University of Colorado Denver, PO Box 173364, Denver, CO 80217, USA

**Keywords:** seabird, penguin, animal-borne camera, predator–prey, confusion effect, indicator species

## Abstract

Predator dietary studies often assume that diet is reflective of the diversity and relative abundance of their prey. This interpretation ignores species-specific behavioural adaptations in prey that could influence prey capture. Here, we develop and describe a scalable biologging protocol, using animal-borne camera loggers, to elucidate the factors influencing prey capture by a seabird, the gentoo penguin (*Pygoscelis papua*). From the video evidence, we show, to our knowledge for the first time, that aggressive behavioural defence mechanisms by prey can deter prey capture by a seabird. Furthermore, we provide evidence demonstrating that these birds, which were observed hunting solitarily, target prey when they are most discernible. Specifically, birds targeted prey primarily while ascending and when prey were not tightly clustered. In conclusion, we show that prey behaviour can significantly influence trophic coupling in marine systems because despite prey being present, it is not always targeted. Thus, these predator–prey relationships should be accounted for in studies using marine top predators as samplers of mid- to lower trophic-level species.

## 1. Introduction

Studies in the marine realm which focus on predator–prey relationships [1–3] face the challenge that simultaneous sampling of both higher trophic order predatory species and their prey is often logistically and financially difficult. Thus, a common approach is to use proxies of prey availability, whereby the level of focus on predator–prey relationships relates to usage of various habitat components within the home range (third-order selection, [4]). This has been achieved using technology such as animal-borne tracking devices and either trawls or acoustic monitoring, to investigate predator and prey distribution, respectively [2,3]. However, while some studies have found concordance between predator and prey distribution [3], others have yielded inconclusive results when relating demographic parameters, distribution and dietary composition of predators to the availability and abundance of prey [2,5,6]. For example, a recent study tracked two penguin species (Adélie (*Pygoscelis adeliae*) and gentoo (*Pygoscelis papua*) penguins) using Argos satellite tags and time-depth recorders, and obtained near real-time distribution of prey fields using autonomous underwater vehicles. While krill aggregation data were not available for every penguin dive, investigators were unable to fully determine whether dense or diffuse aggregations of Antarctic krill (*Euphausia superba*), or species-specific penguin behaviours, drove the observed vertical segregation between penguin species [2]. This means *in situ* studies which can provide empirical evidence at the individual level, about the actual procurement of food items from those available at that site (fourth-order selection) [4], should greatly enhance our understanding of a predator's foraging ecology.

Intrinsic factors, including variable energetic requirements associated with self-maintenance and reproduction, and extrinsic factors, such as anti-predator behaviour employed by prey target species, are known to influence prey selection in terrestrial predators [7,8]. For marine diving predators, such as penguins, there is however limited knowledge regarding how both predator and prey may influence the success of capture [9–12]. Furthermore, penguins have been deemed sentinels of the marine environment [13], with various seabird associated proxies, such as behavioural and demographic measurements, potentially indicating the state of the marine environment [5,6,14]. Therefore, there is a pressing need to better understand behavioural interactions between seabirds and their prey, and the role these upper trophic-level predators might play as samplers of mid to lower trophic-level species.

Understanding predator–prey interactions ideally requires direct observation, which is now feasible for penguins owing to advances in animal-borne camera loggers [15]. The characteristics of gentoo penguin foraging behaviour, specifically the fact that they undertake relatively short foraging trips, make them a well-suited study species for camera deployments. Recent dietary studies at the Falkland Islands, based on stomach content analysis, found that each bird typically feeds consistently during a trip on the same prey items at a given colony and during a specific breeding period [16,17]. Furthermore, gentoo penguins are primarily diurnal, inshore foragers, seldom travelling farther than 30 km from their breeding colony [18]. Therefore, while video cameras have limited recording capacity, the footage obtained should offer valuable insight towards their general foraging behaviours. Thus, the aim of this study was to understand fine-scale predator–prey interactions for gentoo penguins at the Falkland Islands, using animal-borne camera loggers. Furthermore, we develop a widely applicable, freeware protocol, scalable across other studies which require detailed annotation and interpretation of large quantities of video data.

## 2. Material and methods

**Figure 1.**
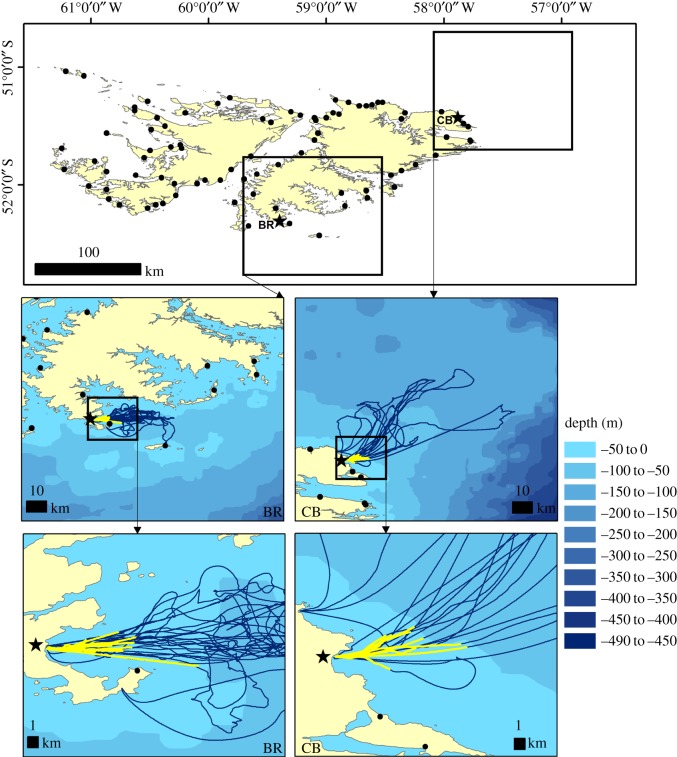
Gentoo penguin colonies (black dots) of the Falkland Islands (top panel), including the two study colonies (stars), Bull Roads (BR) and CB Cow Bay (CB). Tracks (*n*_BR_ = 13, *n*_CB_ = 9) in blue (middle and bottom panel) indicate foraging paths of instrumented birds which had valid GPS data, while yellow overlays indicate the period of time while cameras were recording.

We studied gentoo penguin foraging behaviour during the guard period of chick rearing in December 2013. Thirty-eight birds were sampled from two colonies at the Falkland Islands, Bull Roads (BR) (52.3096° S, 59.3896° W) and Cow Bay (CB) (51.4288° S, 57.8703° W), each with approximately 1236 and 1821 breeding pairs, respectively [19] (figure 1). We chose these colonies because birds depart and return from the sea using a single location, and the colonies are over 500 m away from the shore line. Therefore, birds could be captured without disturbance at the colony. At both colonies, birds typically depart early in the morning (05.00–07.00) for a foraging trip. The cameras used in the study could record for up to 90 min and began recording from the moment they were switched on. Therefore, adult birds were caught while heading to the sea. We chose birds that had a visible brood patch and signs of sitting on a nest. A key sign was to look for fouled birds as nests were typically built from scrub bush material, diddle-dee (*Empetrum rubrum*), on peaty soil. Furthermore, the close proximity of birds within a colony often meant that a nesting individual would be fouled by nearby birds. During instrument deployment, birds were given a unique mark on the breast feathers, to allow for their identification upon return from the sea, using a green, temporary, waterproof wax marker (ROTO.STIK, Sheepman Supply Co.). We recaptured birds after a single foraging trip by maintaining a continual watch of the sea exit point, until 23.00 daily. Upon recapturing, devices were removed, birds were weighed, and bill length and bill depth recorded. We later searched for sampled birds in the colony based on their unique mark, allowing us to confirm the breeding status.

The camera deployments occurred as part of an ongoing study where birds were fitted with a: CEFAS G5 time-depth recorder (TDR; CEFAS Technology Ltd, Lowestoft, UK), CatTraQ GPS logger (Catnip Technologies) and custom waterproofed Replay XD 1080 HD camera (Stable Imaging Solutions, LLC, USA) (electronic supplementary material, figure S1). Devices were set to record at 1 s intervals, 1 min intervals and 30 frames per second, respectively. The cumulative mass of devices was 172.7 g, accounting for ≈2.7% mass of the instrumented birds and ≈6% of birds’ cross-sectional surface area. Devices were secured to the birds using overlapping layers of waterproof adhesive TESA^®^ tape (Beiersdorf, AG, GmbH, Hamburg, Germany), with the tape ends sealed using cyanoacrylate glue (Loctite 401^®^). Securing units this way ensures the plumage is left untarnished following device removal.

A standard protocol for the annotation and quantification of video data derived from animal-borne camera loggers does not yet exist. Thus, we developed a protocol using freeware. First, we converted video from .MOV to .AVI with MPEG Streamclip (v. 1.2) [20], so that, secondly, video could be annotated using Solomon Coder (v. 16.06.26) [21]. We recorded 11 main categories of observations (detailed in the electronic supplementary material). Specific to this study are categories that relate to prey, and intra- and interspecific interactions. Furthermore, the bird's orientation in the water column was recorded, based on physical features (sea surface/sea floor) and the changing light-intensity levels, evident when the bird was ascending or descending. Also, when birds were foraging along the sea floor, we recorded whether birds used upward or downward strikes of the head during attempted prey captures (APC). It was not always feasible to determine if prey were consumed or not. Therefore, we defined an APC as the clearly distinguished moment of a bird actively raising and striking its head towards the prey item until the moment its head returned to a neutral position, after the bird may have either been successful in capturing the item or not.

Prey size was estimated by comparing it to penguin bill size at the moment the prey was adjacent to the bill, thus limiting the effect of unknown distance which could confound this measurement. The estimation of prey size allowed us to categorize prey both by type and broad size class (e.g. small versus large). When prey were aggregated, we noted whether aggregations were loosely or tightly clustered. Loose aggregations were characterized as those which had obvious space between prey items and where one could clearly see through the aggregation during the entire period that the bird was approaching. Tightly aggregated prey were characterized by having no obvious space between prey items and where one could not see through the aggregation during the bird's approach. In line with our freeware protocol, we used custom codes in R 3.1.2 [22] to determine, from the annotated video files, the recording duration, number of APCs and what orientation birds occurred in, and the number of interactions with conspecifics and heterospecifics. We extracted unique behavioural events (still images) by frame number, using the freeware FFmpeg (v. N-82324-g972b358) [23].

Data from TDR and GPS devices were processed as part of the ongoing study, which allowed for the visualization of where along the foraging path footage was recorded from. Specifically, data from TDR devices were processed with the ‘diveMove' package [24]. GPS data were first filtered for erroneous locations using the *speedfilter* function (‘trip' package, [25]), based on the algorithm by McConnell *et al*. [26], when the average transit speed between them was greater than 8 km h^−1^ [27]. Furthermore, the diving nature of penguins results in intermittent positional fixes. Therefore, the filtered data were processed using a continuous-time correlated random walk model (implemented in the ‘crawl' package, [28]) to generate the most probable path used by a bird, through simulation of 100 possible tracks. The filtered data were matched to the camera annotations using the ‘interp1’ function of the ‘signal' package [29]. As data from the three devices were prone to clock drift, data were visually aligned using the Ethographer extension in IGOR Pro (WaveMetrics, Inc.) [30].

Data are presented as mean and standard deviation, unless stated otherwise. The electronic supplementary material provides the camera annotations and R script from the study [31]. Furthermore, we also provide a stepwise example of software use, example data files and custom R script, which also shows how to merge data from multiple tags.

## 3. Results

We obtained suitable footage from 14 and 17 birds at BR and CB, respectively, yielding a total of 35.6 h of footage which was recorded from the beginning of foraging trips (figure 1). In the remaining cases, three birds were not recaptured despite a week of continuous observation for birds post-deployment. Thus, we suspect these to have been non-breeders as gentoo penguins guarding chicks rarely forage over multiple days before returning to the nest [18]. The other four birds were recaptured, but only entered the water after the cameras had ceased recording. On average, the first 69 (±12.6) min of a trip were recorded and all birds, apart from one, had APCs within the video recording.

APCs involved foraging on seven different prey types, with an average of 52 (0–284, median/range) and a total of 1932 individual APCs being identified for each bird and across all birds, respectively (electronic supplementary material, movie S1, shows examples of each prey type observed during APCs). The seven types of prey involved in the APCs included lobster krill (*n* = 599, *Munida* spp*.*), small fishes (*n* = 375, probably juvenile rock cod, either *Patagonotothen tessellata* or *Patagonotothen ramsayi*, less than 30–40 mm fish standard length (tip of the snout to posterior end of the last vertebra)), larger fishes (*n* = 4, unidentified, greater than 70 mm fish standard length) and adult squid species (*n* = 4, probably Patagonian squid (*Doryteuthis gahi*)). We also observed 78 APCs on two unidentifiable items (item 1, *n* = 27; item 2, *n* = 51) and 872 APCs where birds showed the characteristic head-striking movement of an APC, but no prey item could be observed. It is probable that the majority of these 872 APCs were also for small fishes or possibly, but less probably, the amphipod, *Themisto gaudichaudii,* based on previous dietary studies in the region [16] and the similar characteristic in head strike movement when small fishes were definitively observed (J. M. Handley 2014, personal observation).

Birds did not appear to pursue either lobster krill or small fishes and swam in a uniform fashion using quick strikes of the head to capture prey which were present within their trajectory. When birds clearly missed these prey items (*n* = 109), they did not appear to deviate from their course and continued swimming uniformly. This contrasted with the larger squid and fishes, where it was clear that birds pursued prey. However, these larger items were seldom encountered (*n* = 8).

Based on the orientation of birds evident in the camera footage, birds primarily fed while ascending, followed nearly equally by feeding in the water column where orientation was unclear (pelagic foraging) or with upward strikes of the head while foraging along the sea floor (table 1). Furthermore, for the lobster krill, there were relatively few APCs while foraging along the sea floor (*n* = 9), despite clear evidence in 64 separate events where lobster krill were present on the sea floor. An event was considered from the moment a bird began swimming over a section of sea floor containing lobster krill, until the section ended, and each lasted an average of 2.3 s (0.17–31.4 s, median/range) (electronic supplementary material, movie S1). Rather, APCs on lobster krill occurred primarily by birds attacking single individuals while ascending or foraging pelagically (table 1).

**Table 1.** Orientation of gentoo penguins while feeding on all prey and the two main prey types observed, lobster krill (*Munida* spp.) and small fishes (probably *Patagonothen* spp*.*). (Total number of attempted prey captures (APCs) and percentage are shown.)

penguin orientation	all prey items (%)	lobster krill (%)	small fishes (%)
surface (stationary)	0 (0)	0 (0)	0 (0)
surface (swimming below)	1 (0.1)	0 (0)	0 (0)
descend	26 (1.3)	5 (0.8)	5 (1.3)
sea floor (head down)	65 (3.4)	9 (1.5)	4 (1.1)
sea floor (head up)	479 (24.8)	4 (0.7)	107 (28.5)
pelagic	525 (27.2)	182 (30.4)	86 (22.9)
ascend	836 (43.3)	399 (66.6)	173 (46.1)
total	1932 (100)	599 (100)	375 (100)

**Figure 2.**
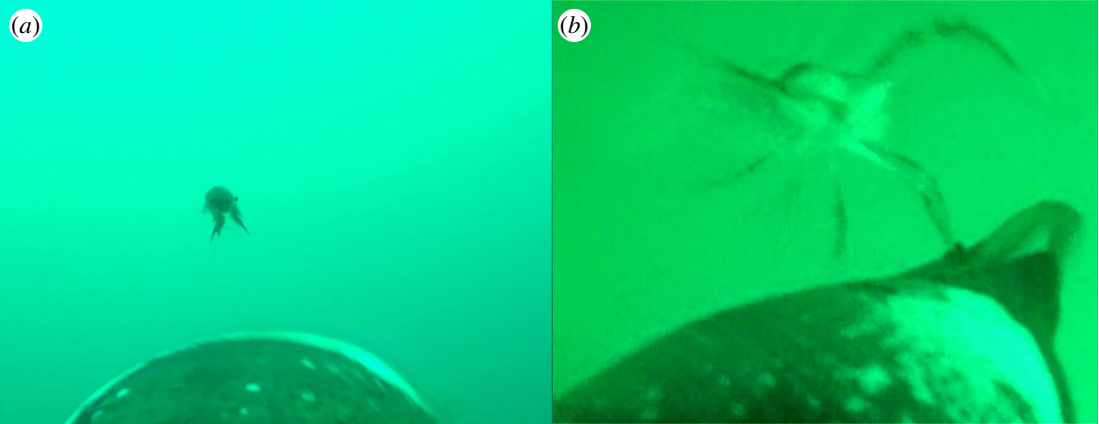
Lobster krill *Munida* spp. (*a*) Defensive position—pincers open—as the bird heads towards it. (*b*) Lobster krill is attacking the bird with pincers during an attempted prey capture (APC). In both these instances, birds were unsuccessful in capturing the lobster krill.

**Figure 3.**
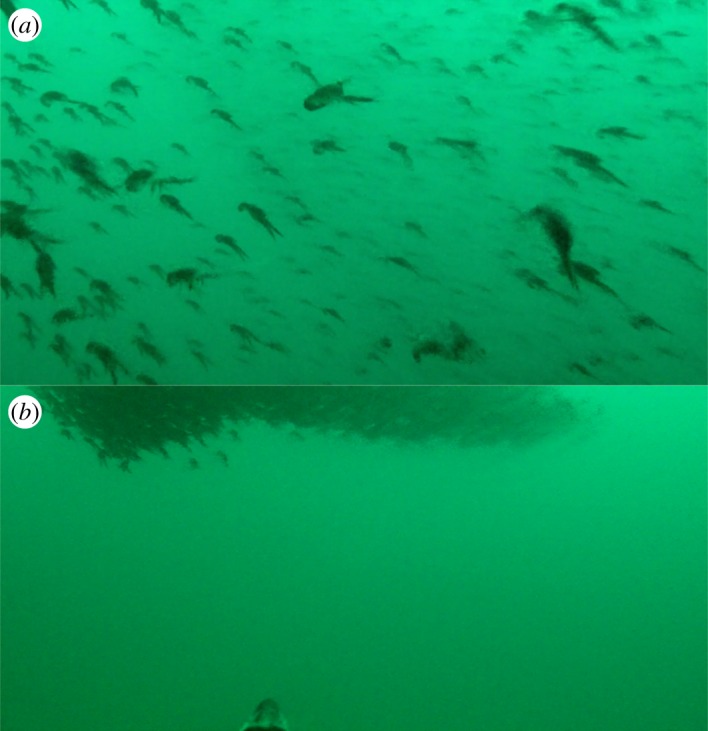
Gentoo penguins were observed to feed off (*a*) loosely clustered swarms of lobster krill (*n* = 16); however, the birds did not feed off (*b*) tightly clustered swarms (*n* = 28).

There were 29 events, involving 10 different birds, where we observed individual lobster krill avoiding capture by actively defending themselves with their pincers (figure 2; electronic supplementary material, movie S2). Five birds also encountered lobster krill swarms (*n* = 44) during their foraging trip. Sixteen of these swarms looked to be loosely aggregated, and in these instances birds fed from the periphery. One bird swam directly into a loosely aggregated swarm and captured lobster krill. However, for the other 28 swarms, in which lobster krill appeared tightly clustered, birds headed towards them but did not feed off the swarms (figure 3; electronic supplementary material, movie S3).

There was no evidence of birds hunting prey cooperatively (e.g. more than one penguin foraging on the same prey patch), as birds from both colonies had negligible interactions (percentage of trip time), with both conspecifics (BR = 0.43%[0.86], CB = 3.66%[0.86]) and other penguin species (BR = 0%[0], CB = 0.13%[0.17]). When interactions did occur, they appeared to be either chance encounters with the subject bird either ignoring other individuals or following them briefly (electronic supplementary material, movie S4).

## 4. Discussion

We provide, to our knowledge, the first evidence of a reduction in foraging success for penguins attributable to two anti-predator tactics used by prey: active defence by individuals, and group formation. This highlights a caution for marine predator studies assuming a direct relationship between relative prey availability and dietary composition. Thus, as has often been recognized in terrestrial systems, the context in which prey and predator find themselves must be considered [32,33].

A key consideration in biologging studies is the tag affect. For penguins, there are mixed results regarding the degree to which birds are affected, either neutrally or negatively [34–36]. Based on wind tunnel tests looking at drag on various species, it is clear that numerous aspects should be considered when looking at the effect of a tag on a diving marine predator, such as tag cross-sectional area, average swimming speed, prey capture methods and duration of tag deployment [37,38]. Therefore, while we did not measure the effects of tags on the behaviour of the individuals in our study directly, we expect tag effects on the birds, and their prey capture ability, to be negligible for the following reasons: (i) the typical prey capture method by gentoo penguins did not involve birds actively pursuing prey; the same type of prey readily observed in dietary studies [16]; (ii) even when gentoo penguins did actively pursue prey, we observed them to be successful in capturing large squid which required pursuit; and (iii) tags were only deployed for a single foraging trip, thereby minimizing possible long-term effects on fitness.

The aggregation of lobster krill into swarms appeared to have an impact on whether gentoo penguins captured these prey items or not. Aggregating prey can reduce susceptibility to predation through attack dilution, increased overall vigilance, communal defence and predator confusion [8]. Disentangling which one, or combination, of these mechanisms may drive swarming behaviour in lobster krill is challenging. However, as birds typically targeted individual lobster krill or those on the periphery of swarms that were not as tightly clustered, this interaction by penguins to swarms of lobster krill lends support to these predators being influenced by communal defence and the confusion effect. The confusion effect arises when prey behaviour limits the ability of a predator to single out prey items from tightly packed groups which present a greater visual barrier; as has been documented for a variety of predators such as invertebrates, fishes and other birds [39,40]. More recently, the first *in situ* observations from African penguins (*Spheniscus demersus*) reaffirm this, as fish separated from the shoal were most likely to be caught by the birds [11].

Regarding communal defence, while we could not observe this directly from the video footage, the attacks observed from individual lobster krill mean that it is likely each swarm constitutes multiple lobster krill defending themselves from attack. Therefore, birds must consider the trade-off between the short-term gain in energy versus the possible long-term reduction in foraging efficiency should the bird become injured. For many species, where individuals have sustained sublethal injuries from prey, these individuals are often limited to catch suboptimal prey with the net effect being reduced fitness [41]. Clearly, the method used by gentoo penguins to capture lobster krill and most prey, which involves attacking individual items from below, helps to minimize handling time and capture prey individuals before they can orientate themselves into a defensive position. This might further explain why birds seldom attacked lobster krill on the sea floor. These individuals are probably able to defend themselves better given their orientation, and also size, as larger adults typically aggregate on the seabed [42].

To overcome prey defensive ability and increase the chance of singling out prey in a school, or swarm, predators often use a cooperative hunting strategy [8,43,44]. While group foraging has been observed by gentoo penguins at Antarctic localities [9,45], the camera footage revealed that this was not the case for gentoo penguins at the Falkland Islands. For other penguin species, variable evidence suggests that birds may forage individually or cooperatively [9,46,47]. However, even for those species that show cooperative foraging, they may still be more successful when targeting aggregating prey alone [10]. This appears to be in contrast to a situation where multispecies assemblages attacking grouped prey increased the feeding success of each individual [48]. These studies, however, were not able to consider prey defensive ability. Therefore, our study reinforces that prey ability to avoid predation, and whether predators forage alone or cooperatively, must be considered when exploring broader facets relating to predator–prey dynamics [5,6,14].

Notably, birds did not deviate from their general swimming direction when they missed lobster krill or small fishes. Birds did, however, actively chase after the eight larger prey items; which might indicate that their behaviour is consistent with optimal foraging theory [49,50]. Thus, our anecdotes may indicate that penguins will exert a greater amount of energy when the returns would be higher. This behaviour, and those discussed above, imply that birds may attend to the specific challenges presented by each prey type. Furthermore, gentoo penguins may keep track of potential prey availability within their home range when one considers the ‘predator pass-along effect' [7]. This mechanism is driven by predator movement as a consequence of unsuccessful attacks, and suggests that a predator might spread the risk over many hunting sites to manage prey behaviour, benefiting the predator's long-term energy intake.

While our study highlights a predator–prey interaction for gentoo penguins at only one locality, the use of animal-borne camera loggers provided clear evidence that where there is readily available prey, this may not necessarily be targeted by the predator. Hence, while Antarctic krill cannot defend themselves like lobster krill, our study provides insight into why there may be a mismatch between predator and prey distribution observed for gentoo penguins elsewhere [2]. The implications of our study are that considerations such as the ability of prey to avoid predation, and the degree to which predator and prey interact when in relatively close proximity, must be considered when characterizing dynamic marine systems. Thus, caution must be taken against oversimplifying trophic studies involving marine top predators because we may arrive at naive conclusions when relating demographic parameters or distribution, as well as dietary composition of predators, to the availability and abundance of prey [5,6,14].

## Supplementary Material

Instructions for supplementary data and video captions

## Supplementary Material

Additional method details, and solutions for video analysis

## Supplementary Material

Behavioural Category Descriptors

## Data accessibility

Raw data and additional information uploaded as the electronic supplementary material and in Dryad repository (http://dx.doi.org/10.5061/dryad.5247q) [31].
