# Supplementary material for: Behaviourally mediated predation avoidance in penguin prey: in situ evidence from animal-borne camera loggers
Source: R Soc Open Sci. 2018 Aug 22;5(8):171449. doi: 10.1098/rsos.171449 (PMC6124084; doi:10.1098/rsos.171449)
Supplement: Instructions for supplementary data and video captions [file rsos171449supp1.docx]

**Supplementary material and Dryad repository (DOI: 10.5061/dryad.5247q) file instructions**

**Temporary Dryad link:** <http://datadryad.org/review?doi=doi:10.5061/dryad.5247q>

**Title**

Behaviourally mediated predation avoidance in penguin prey: *In situ* evidence from animal-borne camera loggers

Jonathan M Handley^1^, Andréa Thiebault^1^, Andrew Stanworth^2^, David Schutt^3^, Pierre Pistorius^1^

^1^DST/NRF Centre of Excellence at the FitzPatrick Institute of African Ornithology, Department of Zoology, Nelson Mandela University, South Campus, Port Elizabeth 6031, South Africa

^*^jonorow@gmail.com Tel: +27 (0)83 291 4690 Fax: +27 (0)41 504 2317

^2^Falklands Conservation, P.O. Box 26, Stanley FIQQ 1ZZ, Falkland Islands

^3^University of Colorado Denver, PO Box 173364 Denver, CO 80217 USA

The following files supplement the manuscript, “Behaviourally mediated predation avoidance in penguin prey: *In situ* evidence from animal-borne camera loggers”. The files are either linked directly to the paper, or are stored in a Dryad repository.

**Files linked directly to the paper:**

File: “Handley_RSOS_Supp Material_REVSIED.docx”

- Provides an overview of the analyses and directs users to necessary example files.

File: “Handley_RSOS_Behavioural Category Descriptors.csv:

- Excel document that contains descriptions about each behavioural category used in the below six files, and relevant to the study.

**Files available on the Dryad repository (DOI: https://doi.org/10.5061/dryad.5247q)**

File: “HandleyJM_GNP_CAM_Analysis.R”

- A synthesis of R code used to generate the results presented in the paper, including descriptions of all prey categories.

File: “GNP_FLK_CAM_PreyDetail.Rdata”

- R data file of the annotated camera data used for analyses in the paper

File: “P1_AnimalCam_SuppCode_Merge Trips.R” & “P2_AnimalCam_SuppCode_Feeding events.R”

- R scripts which require the other supplementary files:
  - SpeciesX_Location_2013_DepID21_1of3_Vid.csv
  - SpeciesX_Location_2013_DepID21_2of3_Vid.csv
  - SpeciesX_Location_2013_DepID21_3of3_Vid.csv
  - SpeciesX_Location_2013_DepID24_1of3_Vid.csv
  - SpeciesX_Location_2013_DepID24_2of3_Vid.csv
  - SpeciesX_Location_2013_DepID24_3of3_Vid.csv

File: “P3_AnimalCam_SuppCode_MergeCAMtoTDRandGPS.R”

- R script which gives example of how data from multiple devices can be merged, and requires the supplementary R files:
  - BR14_GNP_FLK_2013_CAM_SuppData.R
  - BR14_TDR_2013.14_GentooFI_ZOCdives.csv
  - BR14_2013-14_GentooFI_bf_track.csv

Short title and captions for movies S1-S4 which are provided in the supplementary material:

Movie S1:

Movie S1, Short title: General footage overview for gentoo penguins

Movie S1 Caption: General overview of footage obtained from animal-borne camera loggers attached to Gentoo penguins at the Falkland Islands. Examples include: Feeding on lobster krill, feeding on large fish, a full dive where the bird feeds on small fish then pursues and captures a squid, a full dive where lobster krill on the seafloor is ignored and unidentified prey items are shown, lobster krill on seafloor being predated upon, and foraging for small fish.

Movie S2:

Movie S2, Short title: Lobster krill defends against predation

Movie S2, Caption: Examples of lobster krill actively avoiding predation by gentoo penguins, by defending themselves with their pincers.

Movie S3:

Movie S3, Short title: Swarms of lobster krill

Movies S3, Caption: Examples of gentoo penguins not feeding and feeding off of large swarms of lobster krill.

Movie S4:

Movie S4, Short title: Intra and interspecific interactions

Movie S4, Caption: Examples of intra and interspecific interactions for gentoo penguins at the Falkland Islands.
